# Novel Ethanol‐Sensitive Mutants Identified in an F3 Forward Genetic Screen

**DOI:** 10.1111/acer.14240

**Published:** 2019-12-17

**Authors:** Mary E. Swartz, Charles Ben Lovely, Neil McCarthy, Tim Kuka, Johann K. Eberhart

**Affiliations:** ^1^ Department of Molecular Biosciences Waggoner Center for Alcohol and Addiction Research Austin Texas; ^2^ Present address: Department of Biochemistry and Molecular Genetics University of Louisville Louisville Kentucky; ^3^ Present address: Dana‐Farber Cancer Institute Harvard Medical School Boston Massachusetts

**Keywords:** Fetal Alcohol Spectrum Disorders, Zebrafish, Alcohol, Genetics, FASD

## Abstract

**Background:**

Fetal alcohol spectrum disorders (FASD) collectively refer to all deleterious outcomes due to prenatal alcohol exposures. Alterations to the face are common phenotypes in FASD. While alcohol exposure is the underlying cause of FASD, many variables modify the outcomes of such exposures. Genetic risk is one such variable, yet we still have a limited understanding of the nature of the genetic loci mediating susceptibility to FASD.

**Methods:**

We employed ENU‐based random mutagenesis in zebrafish to identify mutations that enhanced the teratogenicity of ethanol (EtOH). F3 embryos obtained from 126 inbred F2 families were exposed to 1% EtOH in the medium (approximately 41 mM tissue levels). Zebrafish stained with Alcian Blue and Alizarin Red were screened for qualitative alterations to the craniofacial skeleton between 4 and 7 days postfertilization (dpf).

**Results:**

In all, we recovered 6 EtOH‐sensitive mutants, 5 from the genetic screen itself and one as a background mutation in one of our wild‐type lines. Each mutant has a unique EtOH‐induced phenotype relative to the other mutant lines. All but 1 mutation appears to be recessive in nature, and only 1 mutant, au29, has apparent craniofacial defects in the absence of EtOH. To validate the genetic screen, we genetically mapped au29 and found that it carries a mutation in a previously uncharacterized gene, si:dkey‐88l16.3.

**Conclusions:**

The phenotypes of these EtOH‐sensitive mutants differ from those in previous characterizations of gene–EtOH interactions. Thus, each mutant is likely to provide novel insights into EtOH teratogenesis. Given that most of these mutants only have craniofacial defects in the presence of EtOH and our mapping of au29, it is also likely that many of the mutants will be previously uncharacterized. Collectively, our findings point to the importance of unbiased genetic screens in the identification, and eventual characterization, of risk alleles for FASD.

## Graphical abstract

Genetics plays a role in risk and resilience to Fetal Alcohol Spectrum Disorder (FASD). However, our understanding of the genes involved in this risk is very limited. We used random mutagenesis to identify mutations that sensitize embryos to ethanol exposure. These mutants will provide important insight into the genetics of FASD. Ethanol exposure causes microcephaly and a reduction in the overall size of au32 mutants, compared to wild‐type controls.

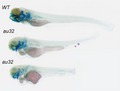

Prenatal ethanol (EtOH) exposure can result in fetal alcohol spectrum disorders (FASD). Phenotypes present in individuals with FASD are highly variable and frequently include alterations to the nervous system and/or face. Numerous variables contribute to the effects of prenatal EtOH exposures, including EtOH dosage, timing of the exposure, and individual genetics (Riley et al., 2011; Streissguth and Dehaene, 1993).

We are gaining an understanding of the genetic risk to FASD. Allelic variants of genes involved in EtOH metabolism have been shown to associate with risk for FASD in humans (Warren and Li, 2005). However, most of our knowledge of the genetic risk for FASD comes from candidate gene studies in animal models. Candidate genes such as members of the Sonic hedgehog, retinoic acid, and nitric oxide pathways have been studied due to the similarities in phenotypes between these pathway mutants and individuals with FAS (Eberhart and Parnell, 2016).

We have previously used available zebrafish craniofacial mutants as candidates to identify and characterize EtOH‐sensitive mutants (McCarthy et al., 2013; Swartz et al., 2014). Our results have identified EtOH‐sensitive mutations that would not have been readily predicted based on phenotypes of individuals with FASD. While we were able to assess the EtOH sensitivity of many mutants, our genetic screens were still biased to genes with known functions in craniofacial development. To fully appreciate the genetics underlying FASD, it is essential to assay gene–EtOH interactions in a more unbiased fashion.

Due to their high fecundity, zebrafish have been used extensively in forward genetic screens to identify genetic pathways mediating development and behavior in an unbiased fashion (Haffter and Nusslein‐Volhard, 1996). Due to the high level of genetic conservation between zebrafish and humans (Postlethwait, 2006; Woods et al., 2005), these screens have provided important insights into human birth defects (Eberhart et al., 2008; Ingham, 2009; Neuhauss et al., 1996; Piotrowski et al., 1996). Importantly, the genetic pathways regulating craniofacial development are exquisitely conserved across vertebrates (Knight and Schilling, 2006). External fertilization makes zebrafish even more useful for screens to identify risk loci for environmental exposures as potential teratogenic agents can be added directly to the fishes’ water.

Here, we demonstrate that zebrafish is a powerful model organism for the unbiased identification of EtOH‐sensitive mutants. Through a small forward genetic screen, we recovered 5 EtOH‐sensitive mutants and an additional background mutant from our wild‐type stocks. The phenotypes of the mutants vary widely, and the facial skeleton of the majority of the mutants develops normally in the absence of EtOH. We demonstrate that at least one of these mutants is in a previously uncharacterized gene. Collectively, our genetic screen will provide new insights into the genetic inputs involved in the variability within FASD.

## Materials and Methods

### Mutagenized Zebrafish Lines

**Figure 1:**
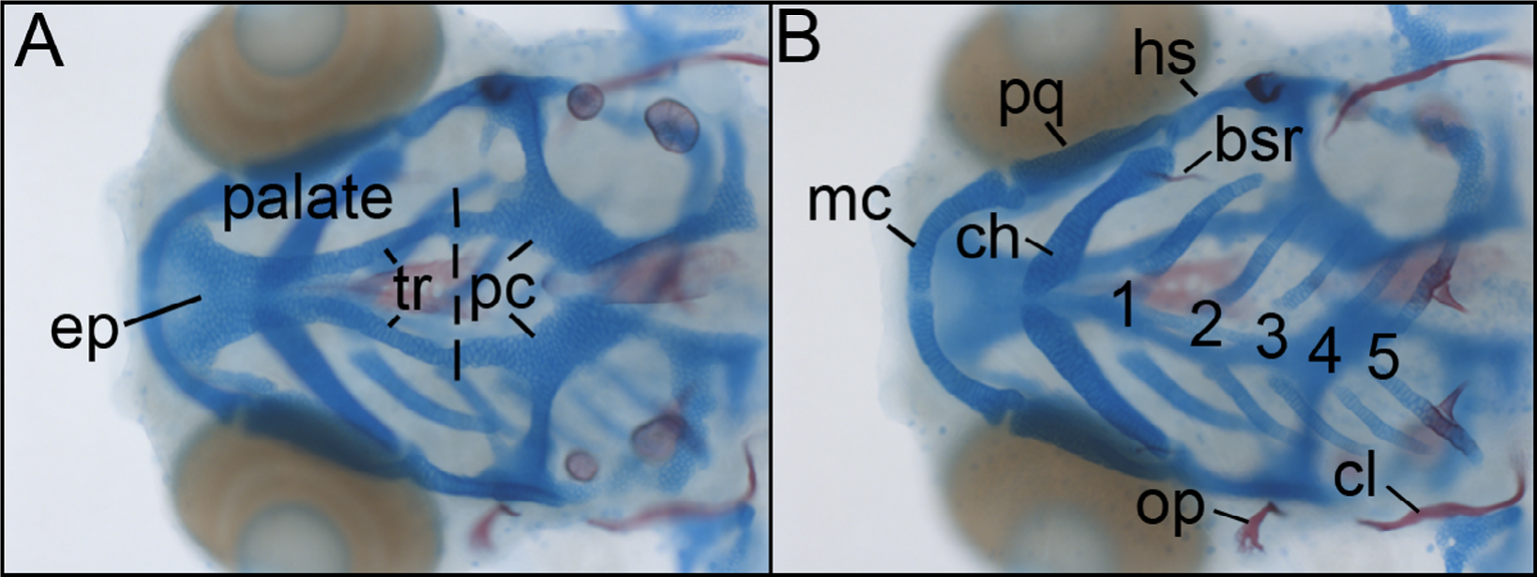
Wild‐type zebrafish craniofacial anatomy. Dorsal (**A**) and ventral (**B**) views of a 5 dpf whole mount Alcian Blue/Alizarin Red‐stained zebrafish. Our forward genetic screen identified EtOH‐sensitive mutants with qualitative alterations to the craniofacial skeleton. (**A**) The neurocranium consists of an anterior, neural crest‐derived palatal skeleton, composed of the trabeculae (tr) and ethmoid plate (ep). The palate connects posteriorly to the mesoderm‐derived parachordal cartilages (pc). (**B**) The viscerocranium consists of 7 segments. The first and second segments have distinct ventral and dorsal skeletal elements at this age. Meckel’s cartilage (mc) and the palatoquadrate (pq) reside ventrally and dorsally, respectively, in the first pharyngeal arch. Ventral and dorsal elements within the second pharyngeal arch are the ceratohyal cartilage (ch) with its associated bone the branchialstegal ray (bsr) and the hyosymplectic cartilage (hs) and opercle bone (op), respectively. The remaining 5 pharyngeal arches house ceratohyal cartilages 1 to 5 (numbered), with the fifth ceratohyal harboring the pharyngeal teeth. The mesoderm‐derived cleithrum (cl) resides just posterior to the pharyngeal arches. Modified from Swartz and colleagues (2014).

Zebrafish were mutagenized with ENU according to established procedures for an F3 forward genetic screen (Solnica‐Krezel et al., 1994). Eye phenotypes obtained from this screen have previously been described (Lee et al., 2012). The same mutagenized stocks were screened for EtOH‐induced facial phenotypes (see Fig. 1 for wild‐type morphology). In all, we screened the offspring from 126 inbred F2 families. For both EtOH‐treated and control conditions, we screened a minimum clutch size of 50 embryos for our initial assessment. Parents of clutches with apparent EtOH‐induced mutations were mated a second time, a minimum of 50 embryos were screened to validate the initial assessment, and lines were established for these validated mutants. All zebrafish were housed and cared for with IACUC approval at The University of Texas at Austin.

### Histological Staining and Imaging

We stained cartilage and bone in 4 to 7 days postfertilization (dpf) zebrafish using a previously described Alcian Blue/Alizarin Red staining protocol (Walker and Kimmel, 2007). Fluorescent Alizarin Red images were performed as described (Eames et al., 2013). Whole mount and flat mount images of histological stains were collected on a Zeiss Axio Imager. Fluorescent images were collected on a Zeiss 710 confocal microscope.

### Genetic Mapping of au29 and Generation of au113 Mutants

Carriers for *au29*, derived in an AB genetic background, were crossed to the WIK genetic background to generate a hybrid stock for genetic mapping. Carriers from this hybrid stock were identified, and their mutant offspring were used for SNP mapping. The DNA from 25 mutant offspring was pooled, and whole genome sequencing was performed by the GSAF at The University of Texas at Austin. Sequence reads were assembled and mapped to the zebrafish genome (ZV9) using MegaMapper (Obholzer et al., 2012). MegaMapper calculated SNP frequency and mapping. To validate the *au29* allele, we generated a second mutation in *si:dkey‐88l16.3* via CRISPR/Cas9, using the Alt‐R CRISPR system (IDT). A gRNA targeting exon 18 (GAGAGAAGCCAGAGCTGCGC) was complexed with Cas9 and injected into 1‐cell stage embryos. P0 fish were backcrossed to wild‐type AB fish and screened for germline transmission of deleterious mutations via RFLP using FspI, which would be disrupted in indel‐containing fish. The nature of the mutations was determined via Sanger sequencing. We selected a 5‐bp deletion for our analyses due to its predicted frame shift.

## Results

### The au26 Allele Is an EtOH‐Sensitive Background Mutation That Disrupts Palatal Development

To initiate our forward genetic screen, we first sought to ensure our wild‐type stocks did not harbor background sensitivity to EtOH. We tested both wild‐type AB and Tubingen strains. We found that our AB stock is resistant to facial alterations caused by exposure to 1% EtOH in the medium (McCarthy et al., 2013; Swartz et al., 2014). Interestingly, we found a portion of clutches obtained from pair‐wise matings of Tubingen fish appeared to have an EtOH‐sensitive background mutation (*n* = 2/11). A mating colony was established from these fish, and through subsequent analyses, we determined that the fish carry a simple recessive mutation causing EtOH sensitivity. We designated this mutant allele *au26*.

**Figure 2:**
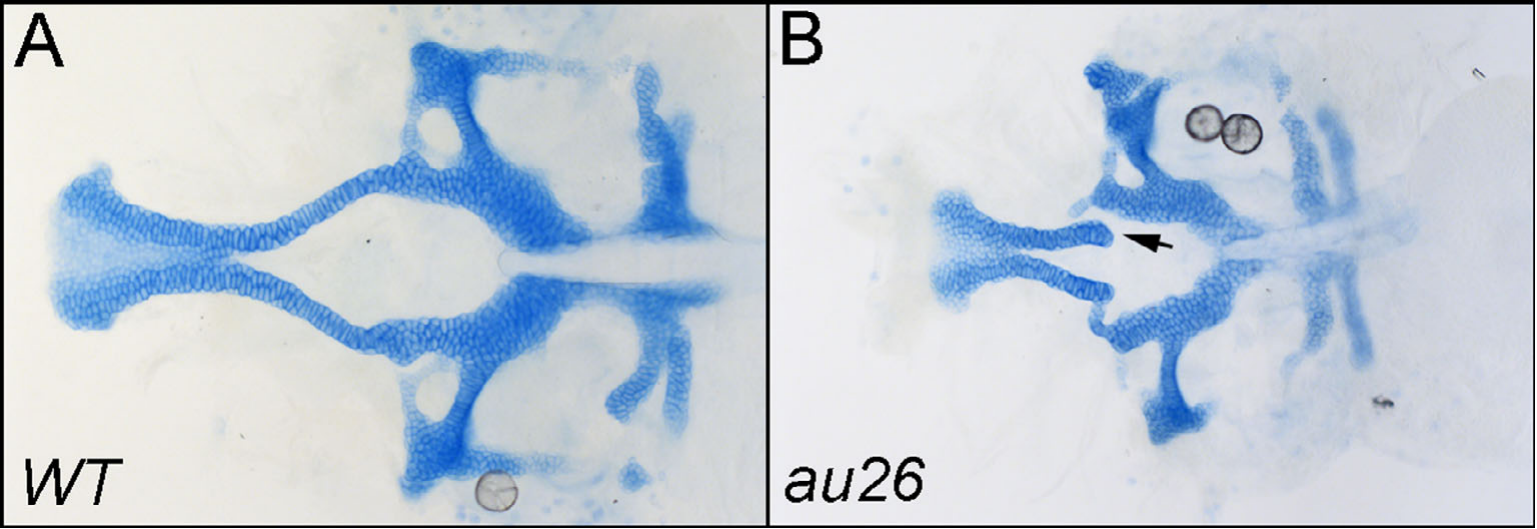
The *au26* mutation results in susceptibility to EtOH‐induced palatal defects. Flat mounts of 5 dpf EtOH‐treated wild‐type (**A**) and *au26* mutant (**B**) zebrafish. The anterior neurocranium is severely reduced in EtOH‐treated *au26* mutants, and the trabeculae fail to appropriately fuse to the posterior neurocranium (**B**, arrow).

We observe no defects in untreated embryos from incrosses of *au26* carriers (not shown). In EtOH‐exposed mutants, the overall size of the craniofacial skeleton at 4 dpf appears slightly smaller (Fig. 2). However, the most striking phenotype in *au26* mutants is a profound defect to the palatal skeleton. The trabeculae are reduced resulting in the ethmoid plate being partially or completely separated from the posterior neurocranium (Fig. 2, arrow). The percentage of mutant phenotypes across clutches varies from 15 to 25%, suggesting that the *au26* mutation is strongly, but perhaps incompletely, penetrant. Once the causative mutation is identified, precise calculations of the penetrance of *au26* can be determined.

### EtOH‐Sensitive Mutants Disrupting Specific Craniofacial Developmental Events

Similar to *au26*, half of the mutants isolated in our forward genetic screen disrupted development of specific craniofacial elements. In EtOH‐treated *au15* and *au28* mutants, skeletal elements of the ventral first arch and dorsal second arch are disrupted, respectively. These effects are only seen in the presence of EtOH; unexposed mutants appear to have normal facial development.

**Figure 3:**
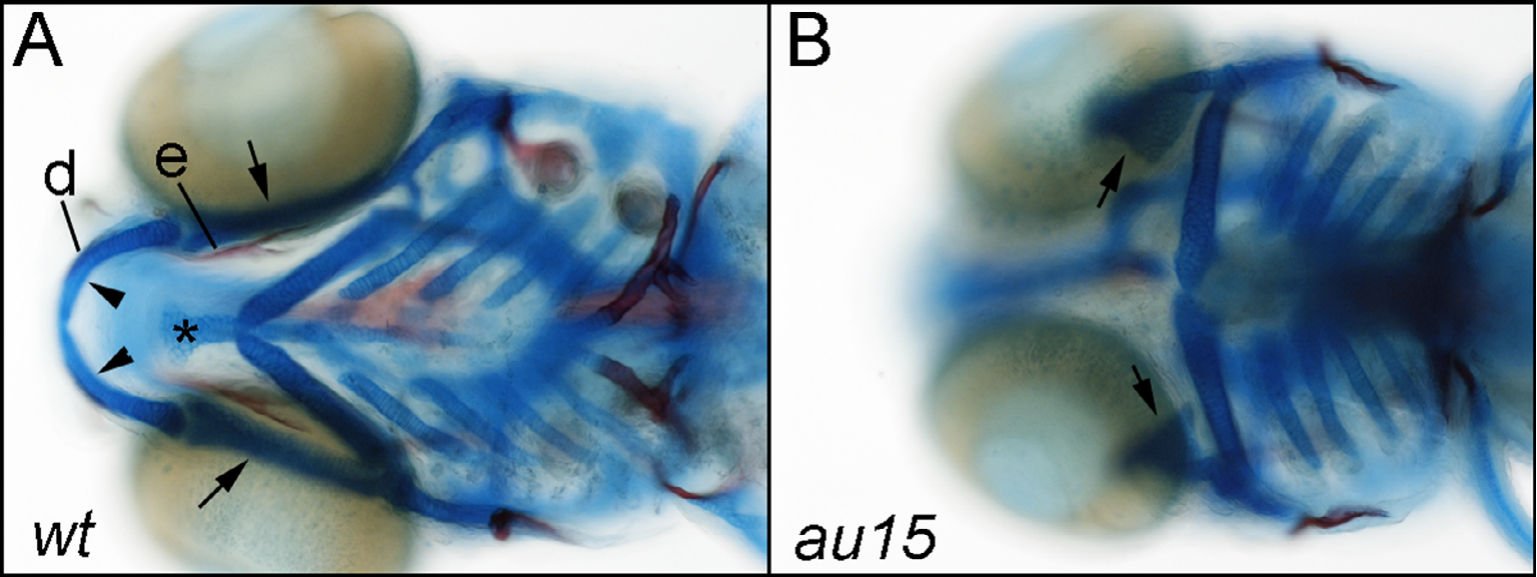
Lower jaw defects in *au15* mutants. EtOH‐treated 5 dpf wild‐type (**A**) and *au15* mutant (**B**) zebrafish. Meckel’s cartilage (**arrowhead**), the dentary (d), and the entopterygoid (e) are lost in *au15* mutants. The palatoquadrate (**arrow**) is reduced in *au15*. In the second pharyngeal arch, the basihyal cartilage (**asterisk**) is also lost in *au15*. Ventral views, anterior to **left**.

In EtOH‐exposed *au15*, there is a severe reduction or loss of the lower jaw. In 5 dpf EtOH‐treated mutants, the dorsal first arch cartilage element, the palatoquadrate, is present albeit reduced (Fig. 3 *A,B*, arrows). However, the ventral cartilage element, Meckel’s cartilage, is lost (Fig. 3 *B*) as are the dentary (d) and entopterygoid (e) bones (Fig. 3 *A*). Within the second pharyngeal arch, the basihyal cartilage (Fig. 3 *A*, asterisk) is lost in EtOH‐treated mutants (Fig. 3 *B*). The mutation appears to behave as a fully penetrant recessive mutation, resulting in 25% mutant phenotypes. We note, however, that in some backcrosses of *au15*, we have observed a reduction in this level of penetrance (from 25% to 10% of a clutch), suggesting that *au15* is subject to genetic modifiers. These currently unknown background modifiers may give important insight into susceptibility to FASD. We observed no defects in clutches that were not exposed to EtOH, suggesting that the normal function of *au15* buffers against EtOH‐induced craniofacial defects.

**Figure 4:**
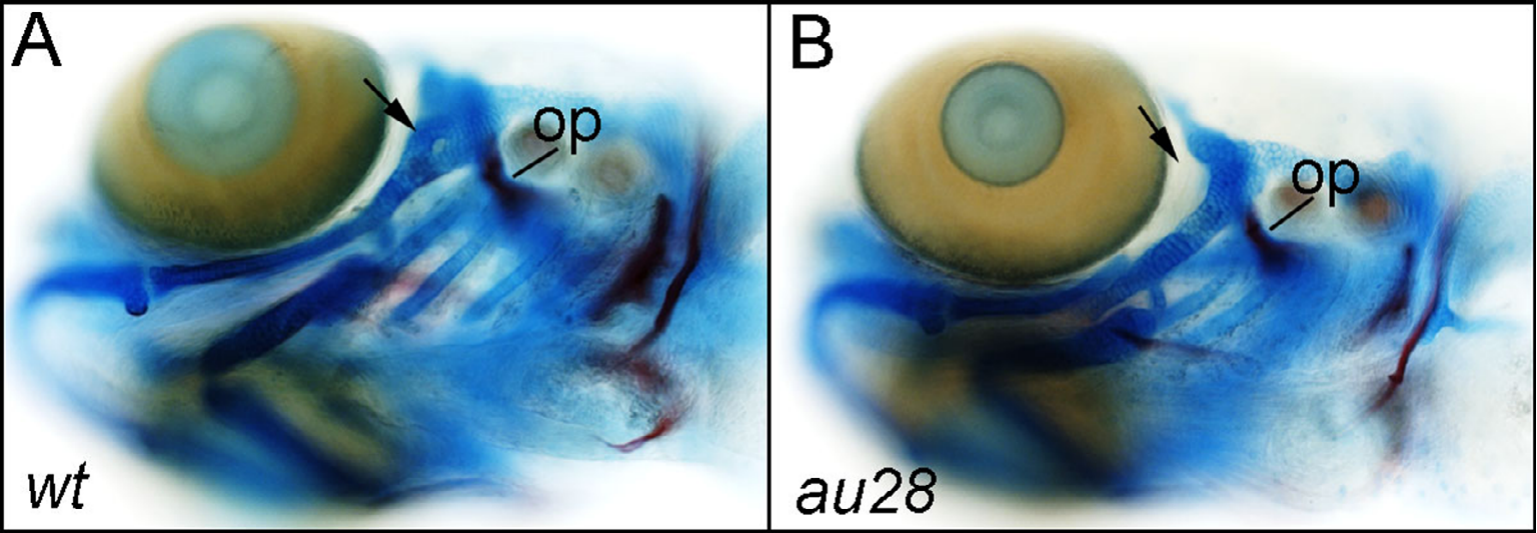
Hyomandibular defects in EtOH‐treated *au28*. The anterior region of the hyomandibular cartilage (**A**, **arrow**) is lost in EtOH‐treated au28 mutants (**B**, **arrow**). The posterior region of the hyomandibular and the associated opercle bone (op) is not affected. Lateral views, anterior to **left**.

In EtOH‐exposed *au28* mutants, there are subtle defects to the hyosymplectic cartilage. The hyosymplectic cartilage consists of the symplectic cartilage rod extending ventrally from the body of the hyomandibular. Based on mutant phenotypes, the hyomandibular can be subdivided into an anterior region, which is sensitive to signals from the adjacent first pharyngeal pouch, and a posterior region, which is insensitive to such disruption (Crump et al., 2004b). The anterior hyomandibular is specifically disrupted in EtOH‐treated *au28* mutants (Fig. 4, arrows). The posterior hyomandibular as well as the opercle bone that attaches to it is present (Fig. 4). Phenotypes range from complete loss to partial loss of the anterior hyomandibular in 1 to 20% of the clutch, respectively. Thus, the *au28* allele is likely incompletely penetrant with variable expressivity.

### Mutants Broadly Disrupting Craniofacial Development

The remaining 3 mutants isolated in our analyses have more broad effects on the craniofacial skeleton. Extensive loss of bone is observed in EtOH‐treated *au27*, and cartilage is broadly disrupted in *au32* and *au29* mutants.

**Figure 5:**
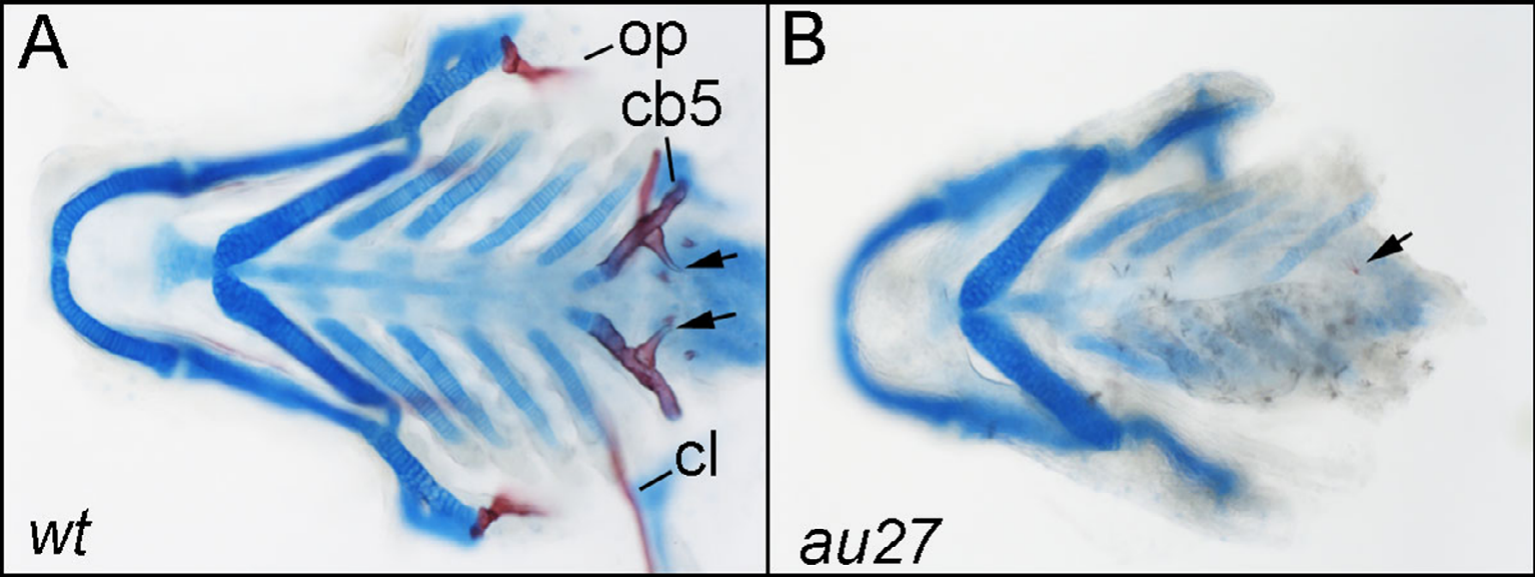
Bone loss in EtOH‐treated *au27* mutants. Flat mounted viscerocrania from wild‐type (**A**) and *au27* mutant (**B**) zebrafish. There is an overall reduction in the size of craniofacial cartilages in *au27* mutants. More striking is the near complete loss of craniofacial bone. Only a tiny remnant of the pharyngeal teeth (**arrows**) remain in mutants. Op, opercle; cb5, ceratobranchial #5; cl, cleithrum. Anterior to the **left**.

**Figure 6:**
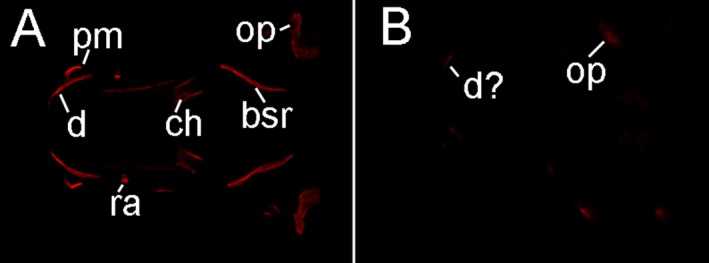
Bone loss in *au27* mutants is not due to a developmental delay. Relative to EtOH‐exposed wild‐type fish (**A**), ossification in *au27* mutants is greatly diminished at 7 dpf. Pm, premaxilla; d, dentary; ra, retroarticular; ch, ceratohyal; op, opercle; bsr, branchiostegal ray. Ventral view, anterior to the **left**.

Similar to the other mutants obtained in this forward genetic screen, *au27* mutants only display defects when exposed to EtOH. At 5 dpf, the entire craniofacial skeleton is slightly smaller in EtOH‐treated *au27* mutants. More striking, however, is the severe reduction in the amount of bone (Fig. 5). There is loss of neural crest‐derived bone (the opercle and the ossification of the fifth ceratobranchial along with its associated teeth) as well as mesoderm‐derived bone (the cleithrum; Fig. 5 *B*). To determine whether this reflected a loss of bone *per se* or a delay in bone differentiation, we analyzed mutants at 7 dpf with a more sensitive live Alizarin Red staining procedure. Confocal imaging of ventral bones demonstrates that the ossification of the ceratohyal and the premaxilla, dentary, opercle, retroarticular, and branchiostegal ray bones had formed appropriately in untreated and EtOH‐treated wild‐type fish (Fig. 6 *A*). However, EtOH‐treated *au27* mutants lack nearly all Alizarin Red staining (Fig. 6 *B*). These phenotypes appear in 25% of fish within a clutch. Thus, we conclude that *au27* is a recessive mutation that sensitizes fish to bone loss following EtOH exposure.

**Figure 7:**
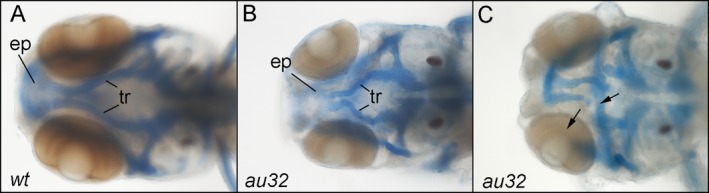
Phenotypic classes of *au32* mutants. Relative to wild‐type fish (**A**), the majority of EtOH‐treated *au32* individuals have severe reductions to the facial skeleton (**B**). In a small percentage of *au32* individuals, there are gaps in the trabeculae and/or Meckel’s cartilage (**C**, **arrows**). Ep, ethmoid plate; tr, trabeculae.

**Figure 8:**
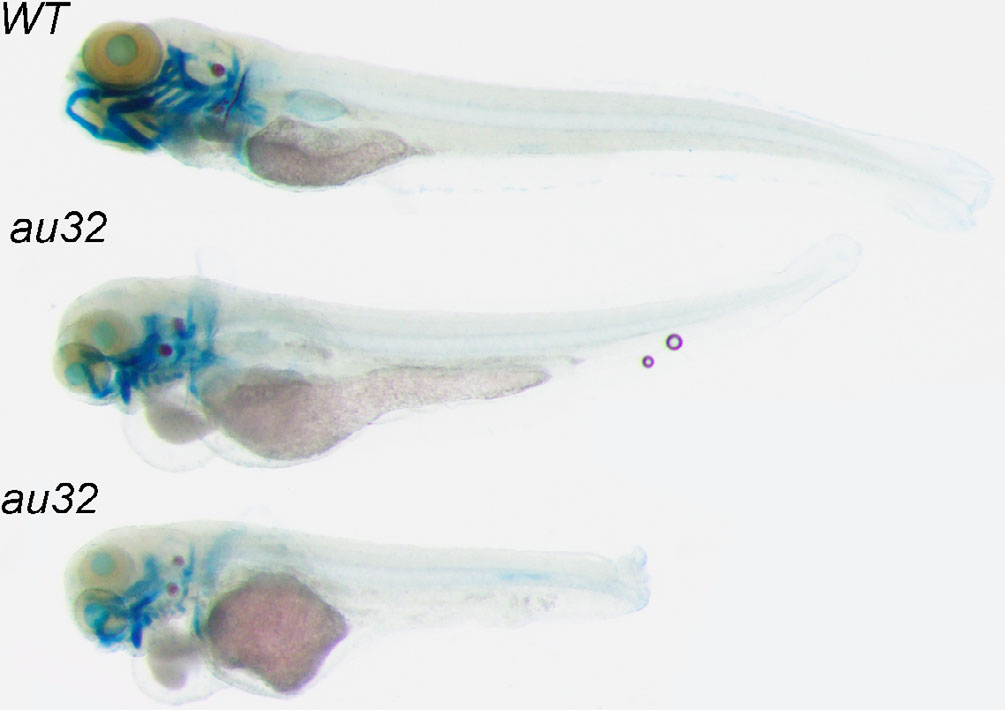
Whole body defects in *au32* mutants. Those *au32* mutants with more profound craniofacial defects also display marked reductions in the overall body axis (*au32*, **bottom**) while less severe *au32* mutants (*au32*, **top**) have a more normal body axis.

Mutants for *au32* also appear normal in the absence of EtOH. In EtOH‐treated clutches of *au32*, just under 50% of fish appear normal in the presence of EtOH, strongly suggesting that *au32* is a partially dominant EtOH‐sensitive allele. The phenotypes of affected zebrafish are variable but fall within 3 major phenotypic classes. The largest phenotypic class (32%) consisted of zebrafish with microphthalmia, cardiac edema, and reduced cartilage (Fig. 7 *B*). Roughly 14% of EtOH‐treated fish were dead or dying by 4 dpf. In addition to microphthalmia, edema, and reduced cartilage, the trabeculae and/or Meckel’s cartilage were absent or greatly reduced in about 6% of fish (Fig. 7 *C*, arrow). Based on these proportions, it is not clear that there is a straightforward genotype–phenotype correlation in EtOH‐treated *au32* fish, as we observed with EtOH‐treated *pdgfra* mutants and heterozygotes (McCarthy et al., 2013). These more severely affected fish also had an overall reduction to the body axis and appeared very unhealthy (Fig. 8, bottom). Thus, it is possible that the early embryonic lethal/severe craniofacial defects associate with homozygous mutants and heterozygosity associates with microphthalmia, cardiac edema, and reduced cartilage. Identification of the causative mutation will be necessary to determine if any such correlation exists.

**Figure 9:**
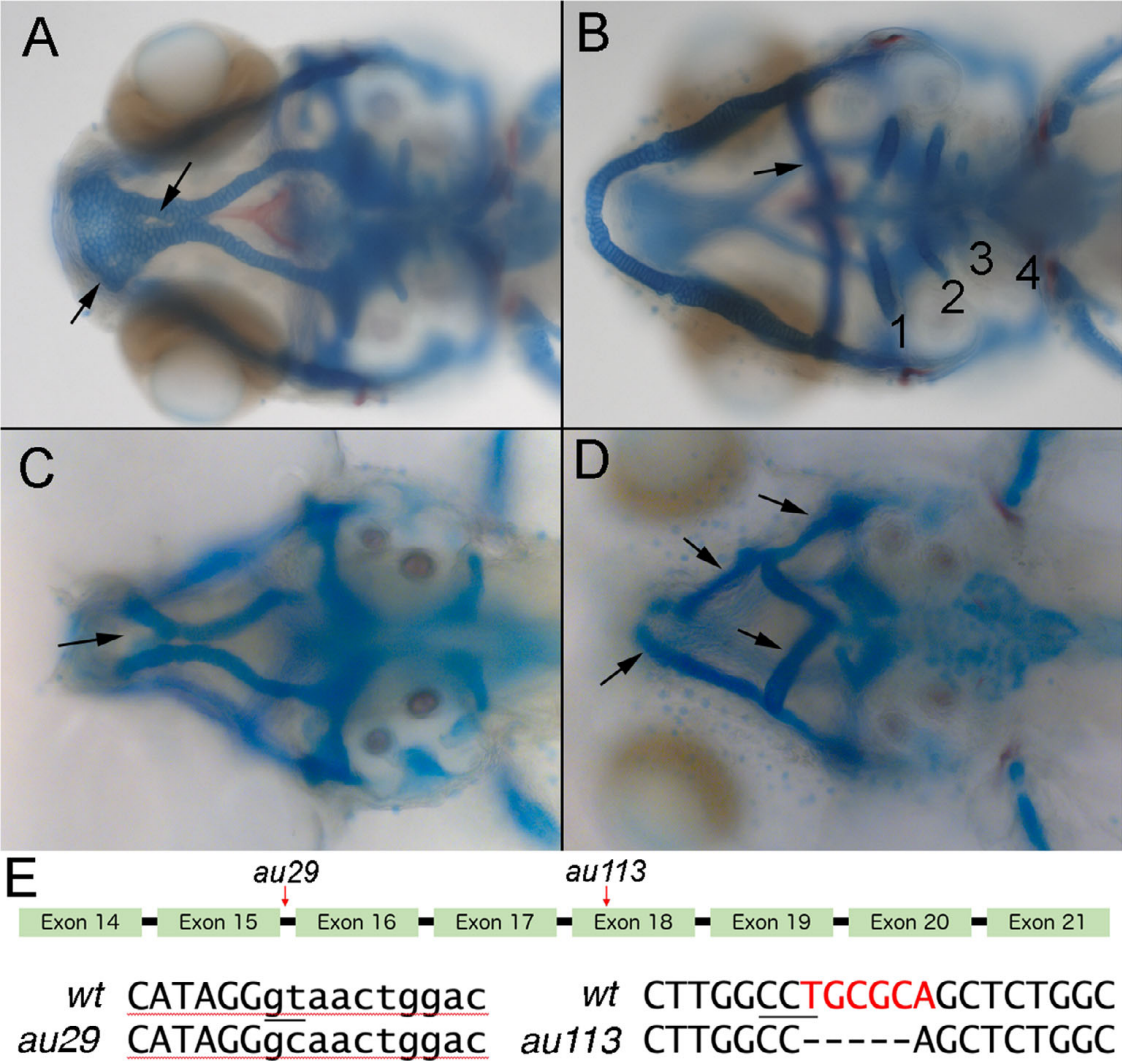
Craniofacial defects in *au29* mutants. In untreated *au29* mutants (**A**, **B**), there are reductions to the ethmoid plate in the neurocranium (**A**, **arrows**). Within the viscerocranium, the ceratohyal cartilages (**arrow**) fail to project anteriorly and the number of ceratobranchial cartilages (**numbered**) is reduced (**B**). EtOH exposure dramatically exacerbates these phenotypes (**C**, **D**). The ethmoid plate is greatly reduced in EtOH‐treated *au29* mutants (**C**, **arrow**; the eyes have been dissected away to more clearly visualize the cartilage). (**E**) Schematic representation of the *au29* and *au113* alleles. Schematic of exons 14 through 21, of 24 predicted exons, is shown with the location of *au29* and *au113* noted. The splice donor of exon 15, underlined in wt sequence, is mutated in *au29*. A 5‐bp indel is present in *au113*. The PAM sequence is **underlined** in the wt sequence, and the FspI site used for genotyping is shown in **red**.

The last mutant from our forward genetic screen, *au29*, is unique among the mutants identified in this screen in that mutant phenotypes are apparent in the absence of EtOH. The size of the eye is notably reduced in untreated *au29* mutants (Fig. 9 *A,B*). Additionally, there are slight disruptions to the ethmoid plate (Fig. 9 *A*, arrow), the angle of the ceratohyal is altered (Fig. 9 *B*, arrow), and the number and size of the ceratobranchial cartilages are reduced (Fig. 9 *B*). Following EtOH exposure, these mutants have profound craniofacial defects. The ethmoid plate is severely reduced (Fig. 9 *C*, arrow). EtOH‐treated mutants lose most of the ceratobranchial cartilages (Fig. 9 *D*). Additionally, the first and second arch cartilage elements are severely reduced (Fig. 9 *D*, arrows). Mutant phenotypes are generated in 25% of fish within a clutch, suggesting that *au29* is a fully penetrant recessive mutant. Given that the EtOH‐induced phenotypes present in our mutants have not been previously described, our collective findings suggest that forward genetic screens are an efficient way to identify gene–EtOH interactions.

### A Novel Member of the LRP Family Is Mutated in au29

Given the uniqueness of the phenotypes in our mutants, it is likely that they harbor mutations in genes not previously characterized in EtOH teratogenesis. To test this, we selected 1 allele, *au29*, on which to perform genetic mapping.

We used MegaMapper for genome assembly as well as variant calling and mapping in *au29* relative to the reference genome. This positioned *au29* on chromosome 10. SNPs predicted to be deleterious in *skor2*, *spp1*, and *si:dkey‐88l16.3* (an annotated, but uncharacterized gene) were identified within 3 Mb of the peak of homozygosity. Each of these SNPs was predicted to disrupt splice sites. We found that the SNPs in *skor2* and *spp1* were not in splice sites and likely represent miscalls by MegaMapper. However, we verified a T to C donor splice site mutation in intron 18 to 19 of *si:dkey‐88l16.3* (Fig. 9 *E*). Using PCR and Sanger sequencing on a separate group of *au29* mutants, we determined that the mutation was homozygous in *au29* mutants but not wild‐type zebrafish. To verify that *au29* is a mutation in *si:dkey‐88l16.3*, we generated a CRISPR/Cas9 allele (*au113*, Fig. 9 *E*). The phenotype of *au113* mutants phenocopies those observed in *au29*. Furthermore, we crossed an *au29* carrier and an *au113* carrier and screened a clutch of 200 embryos. We found that approximately 25% of the embryos had phenotypes identical to *au29* demonstrating that *au29* and *au113* fail to complement. Thus, we conclude that *au29* is a mutation in a previously uncharacterized gene. The predicted protein structure of *si:dkey‐88l16.3* would place it within the LDL receptor‐related protein (LRP) family, important modulators of cell signaling. Mutant or morpholino‐induced loss‐of‐function phenotypes have been described for 6 other members of the LRP family in zebrafish: *lrp1aa*,* lrp1ab*,* lrp2a*,* lrp4*,* lrp5*, and *lrp6* (Jiang et al., 2012; Pi et al., 2012; Saint‐Amant et al., 2008; Veth et al., 2011; Willems et al., 2015). None of these other Lrp family members have been tested for EtOH sensitivity. Only *lrp5* morphants have been characterized to have facial phenotypes potentially due to altered Wnt signaling (Willems et al., 2015) but the predicted structure of *si:dkey‐88l16.3* is distinct from Lrp5. We are currently characterizing the identity and function of this gene (Kuka, et al., in preparation). It will be of great interest to determine whether the Lrp family is broadly sensitive to EtOH exposure and how *au29* alters signaling within the developing embryos.

## Discussion

Our results are the first vertebrate forward genetic screen used to identify mutants with enhanced susceptibility to EtOH teratogenesis. Most human birth defects are thought to arise from complex interactions between genetics and the environment. Most of the mutants that we have recovered show no obvious deleterious phenotypes in the absence of EtOH. Consistent with this, our recovery rate of approximately 1 mutant/25 mutagenized genomes (4%) is higher than that of a large‐scale zebrafish screen in which 109 mutations disrupting craniofacial development were identified in 3,857 mutagenized genomes (2.8%; Piotrowski et al., 1996; Schilling et al., 1996). Given that the mutagenesis procedure used results in approximately 1.1 deleterious mutations per mutagenized genome (Haffter et al., 1996), it is also likely that a minority of mutations will confer EtOH sensitivity, although our screen was relatively small and may not reflect the true number. Thus, it is likely that many sensitizing genetic loci have yet to be identified through candidate approaches. The success of this forward screen in identifying mutations that enhance the teratogenicity of EtOH demonstrates the usefulness of such screens and should be useful in identifying sensitizing alleles for other teratogens.

Each mutant that we obtained has a unique EtOH‐induced defect, suggesting that they are mutations in different genes; however, genetic mapping of the individual mutants will be required to directly determine the identity of the genes disrupted in these mutants. Furthermore, the majority of these mutants have no apparent deleterious phenotypes in the absence of EtOH, suggesting that many of the mutants may be in novel genes. Our genetic mapping of *au29* demonstrates that our screen has already identified previously uncharacterized mutants. Given that all the other mutants appear normal in the absence of EtOH, it is likely that many of these mutants will also not have been identified in previous genetic screens. For those genes that have been characterized, the EtOH‐induced phenotypes are likely to be unknown.

The mechanism of how EtOH disrupts development in this set of mutants is wholly unknown at this point. It remains possible that some mutations alter the uptake or elimination of EtOH, particularly those mutant more broadly disrupting development. However, the wide range of phenotypes that we observe would suggest that EtOH is disrupting genetic pathways regulating specific developmental events. Therefore, genetic mapping and characterizing these mutants will provide important insights into the genetic pathways that mediate susceptibility to EtOH teratogenesis.

### Insights Into Potential EtOH‐Sensitive Pathways: Palatal Development

The phenotype of *au26* mutants closely resembles that of *gata3* mutants (Sheehan‐Rooney et al., 2013) with a specific disruption to the trabeculae of the posterior palate. This striking phenotypic similarity may suggest that *au26* is: (i) an allele of *gata3*, (ii) a member of the same genetic pathway as *gata3*, or (iii) a member of a parallel pathway that converges on the development of the trabeculae.

Interestingly, *gata3* was in our original candidate‐based screen for EtOH‐sensitive mutants (McCarthy et al., 2013). From this, and subsequent analyses, we know that, unlike *au26*, loss of *gata3* does not sensitize embryos to EtOH teratogenesis. Furthermore, we have performed complementation analyses of *au26* and *gata3* (data not shown). We find that all fish from these crosses appear normal. Collectively, these findings strongly suggest that *au26* is not an allele of *gata3*.

Currently, little is known about the pathways specifically regulating the development of the trabeculae. Disruption of Bmp signaling, via expression of a dominant negative Bmp receptor, can result in specific loss of the trabeculae (Alexander et al., 2011). In mouse, *Gata3* is a target of Bmp signaling (Bonilla‐Claudio et al., 2012). EtOH exposure causes ectopic Bmp signaling in the zebrafish heart (Sarmah et al., 2016) and H9c2 cardiomyoblast cells (Shi et al., 2017). Similarly, EtOH exposure elevates the expression of Bmp4 in cultured rat cranial neural crest cells (Wentzel and Eriksson, 2009). It is possible that the overall level of Bmp signaling is critical for activation of *gata3* and that this is perturbed in EtOH‐exposed *au26* mutants. We are currently characterizing the expression of *gata3* in *au26* and Bmp pathway mutants to test this possibility.

No other signaling pathway has been specifically implicated in trabeculae development. However, both the Sonic Hedgehog and Wnt/PCP pathways are important for palate development (Bush and Jiang, 2012; Eberhart et al., 2006; Kamel et al., 2013; Rochard et al., 2016; Wada et al., 2005) and have pathway members that modulate the risk to EtOH teratogenesis (Eberhart and Parnell, 2016). Once the molecular nature of the *au26* allele is discovered, we will be able to more easily determine which genetic pathway(s) is disrupted in this mutant.

### Insights Into Potential EtOH‐Sensitive Pathways: Endoderm Development

While *au15* and *au28* have distinct phenotypes from one another, disrupting development of the ventral first arch versus dorsal second arch (respectively), both of these phenotypes are observed in mutants disrupting endoderm development. Endoderm is necessary for proper craniofacial development (Crump et al., 2004a; David et al., 2002; Lovely et al., 2016) and provides positional patterning information to the neural crest (Couly et al., 2002). Much work on the effects of EtOH on facial development has focused on the neural crest (Smith, 1997). These mutants may suggest an underappreciated role of EtOH on endoderm development.

The loss of Meckel’s cartilage in *au15* mutants phenocopies *sphingosine‐1‐phospate (S1P) type 2 receptor (s1pr2)* mutants. In *s1pr2* mutants, the appropriate migration of anterior‐most pharyngeal endoderm fails and signaling centers required for lower jaw development are not formed appropriately (Balczerski et al., 2012). We have found that *s1pr2* mutants are not EtOH‐sensitive (Lovely, et al, unpublished) and *s1pr2* and *au15* mutants complement (data not shown). Thus, like *au26* and *gata3*, *au15* and *s1pr2* are EtOH‐sensitive and insensitive mutations, respectively, that generate remarkably similar and highly specific phenotypes. Further characterizations will determine whether *au15* disrupts the development of the anterior endoderm, facial signaling centers, or the neural crest cell response to these signaling centers.

The specific loss of the anterior hyomandibular cartilage in *au28* may be due to alteration of the first pharyngeal pouch or the signals the pouch sends to the neural crest. In *integrin alpha 5* (*itga5*) mutants, an endoderm autonomous requirement of *itga5* results in the subsequent loss of the anterior hyomandibular (Crump et al., 2004b). We have yet to determine whether *itga5* is EtOH‐sensitive, but alcohol exposure has been shown to alter integrin levels in the rat brain, trophoblast cells, and human neurospheres (Rout, 2006; Rout and Dhossche, 2010; Vangipuram et al., 2008). There is no clear association of down‐regulated integrin expression in these studies, rather some integrins are upregulated and some are down‐regulated. It is also unclear whether these alterations reflect direct effects of EtOH on transcription or an indirect effect, such as alterations to the relative proportions of cell subtypes expressing different integrins. Regardless of mechanism, however, the phenotype of *au28* mutants strongly suggests an involvement of endoderm or the neural crest response to endodermal signals.

### Insights Into Potential EtOH‐Sensitive Pathways: Bone and Cartilage Development

There are broad defects to bone development in *au27* mutants and cartilage development in *au29* and *au32* mutants. Well‐established genetic pathways regulate the development and differentiation of these tissues. Given that *au27* mutants have defects to bone derived from both neural crest cells and mesoderm, it likely disrupts differentiation of osteocytes in general. Numerous markers of osteocytes and their progenitors, osteoblasts, exist (Hojo et al., 2017). Analyses of transcription factors, such as *sp7* and *runx2*, as well as extracellular matrix molecules, such as *col10a1a*, will provide insight into which steps of bone development are disrupted in *au27*. Given that the *au27* mutation is lethal in EtOH, our current analyses cannot fully rule out a developmental delay nor can we determine whether later forming axial skeletal elements are disrupted. These phenotypic characterizations will provide a deeper understanding of the nature of the *au27* defect. Craniofacial cartilages are largely of neural crest origin. Thus, analyses of genes within the well described neural crest gene regulatory network (Martik and Bronner, 2017), such as *foxd3*, *snail*, *sox10*, and Dlx family genes, will provide insight into *au29* and *au32* mutants. Additionally, analysis of the genes critical for cartilage development, such as *sox9a* and cartilage‐specific collagens, will provide insights into the genesis of the phenotypes in these mutants.

Collectively, these mutants will provide insights into the risk for FASD. As the mutations are identified, we will be able to thoroughly quantify the phenotypes in these mutants. Our characterizations of the genesis of these phenotypes will elucidate the effects of EtOH on the cell behaviors mediating craniofacial development.

## Conflict of interest

The authors have no conflicts of interest to declare.

## References

[acer14240-bib-0001] Alexander C , Zuniga E , Blitz IL , Wada N , Le Pabic P , Javidan Y , Zhang T , Cho KW , Crump JG , Schilling TF (2011) Combinatorial roles for BMPs and Endothelin 1 in patterning the dorsal‐ventral axis of the craniofacial skeleton. Development 138:5135–5146. 22031543 10.1242/dev.067801 PMC3210495

[acer14240-bib-0002] Balczerski B , Matsutani M , Castillo P , Osborne N , Stainier DY , Crump JG (2012) Analysis of sphingosine‐1‐phosphate signaling mutants reveals endodermal requirements for the growth but not dorsoventral patterning of jaw skeletal precursors. Dev Biol 362:230–241. 22185793 10.1016/j.ydbio.2011.12.010 PMC3265674

[acer14240-bib-0003] Bonilla‐Claudio M , Wang J , Bai Y , Klysik E , Selever J , Martin JF (2012) Bmp signaling regulates a dose‐dependent transcriptional program to control facial skeletal development. Development 139:709–719. 22219353 10.1242/dev.073197 PMC3265059

[acer14240-bib-0004] Bush JO , Jiang R (2012) Palatogenesis: morphogenetic and molecular mechanisms of secondary palate development. Development 139:231–243. 22186724 10.1242/dev.067082 PMC3243091

[acer14240-bib-0005] Couly G , Creuzet S , Bennaceur S , Vincent C , Le Douarin NM (2002) Interactions between Hox‐negative cephalic neural crest cells and the foregut endoderm in patterning the facial skeleton in the vertebrate head. Development 129:1061–1073. 11861488 10.1242/dev.129.4.1061

[acer14240-bib-0006] Crump JG , Maves L , Lawson ND , Weinstein BM , Kimmel CB (2004a) An essential role for Fgfs in endodermal pouch formation influences later craniofacial skeletal patterning. Development 131:5703–5716. 15509770 10.1242/dev.01444

[acer14240-bib-0007] Crump JG , Swartz ME , Kimmel CB (2004b) An integrin‐dependent role of pouch endoderm in hyoid cartilage development. PLoS Biol 2:e244. 15269787 10.1371/journal.pbio.0020244 PMC479042

[acer14240-bib-0008] David NB , Saint‐Etienne L , Tsang M , Schilling TF , Rosa FM (2002) Requirement for endoderm and FGF3 in ventral head skeleton formation. Development 129:4457–4468. 12223404 10.1242/dev.129.19.4457

[acer14240-bib-0009] Eames BF , DeLaurier A , Ullmann B , Huycke TR , Nichols JT , Dowd J , McFadden M , Sasaki MM , Kimmel CB (2013) FishFace: interactive atlas of zebrafish craniofacial development at cellular resolution. BMC Dev Biol 13:23 10.1186/1471-213X-13-23 23714426 PMC3698193

[acer14240-bib-0010] Eberhart JK , He X , Swartz ME , Yan YL , Song H , Boling TC , Kunerth AK , Walker MB , Kimmel CB , Postlethwait JH (2008) MicroRNA Mirn140 modulates Pdgf signaling during palatogenesis. Nat Genet 40:290–298. 18264099 10.1038/ng.82 PMC2747601

[acer14240-bib-0011] Eberhart JK , Parnell SE (2016) The genetics of fetal alcohol spectrum disorders. Alcohol Clin Exp Res 40:1154–1165. 27122355 10.1111/acer.13066 PMC5125635

[acer14240-bib-0012] Eberhart JK , Swartz ME , Crump JG , Kimmel CB (2006) Early Hedgehog signaling from neural to oral epithelium organizes anterior craniofacial development. Development 133:1069–1077. 16481351 10.1242/dev.02281

[acer14240-bib-0013] Haffter P , Granato M , Brand M , Mullins MC , Hammerschmidt M , Kane DA , Odenthal J , van Eeden FJ , Jiang YJ , Heisenberg CP , Kelsh RN , Furutani‐Seiki M , Vogelsang E , Beuchle D , Schach U , Fabian C , Nusslein‐Volhard C (1996) The identification of genes with unique and essential functions in the development of the zebrafish, Danio rerio. Development 123:1–36. 9007226 10.1242/dev.123.1.1

[acer14240-bib-0014] Haffter P , Nusslein‐Volhard C (1996) Large scale genetics in a small vertebrate, the zebrafish. Int J Dev Biol 40:221–227. 8735932

[acer14240-bib-0015] Hojo H , Chung UI , Ohba S (2017) Identification of the gene‐regulatory landscape in skeletal development and potential links to skeletal regeneration. Regen Ther 6:100–107. 30271844 10.1016/j.reth.2017.04.001 PMC6134913

[acer14240-bib-0016] Ingham PW (2009) The power of the zebrafish for disease analysis. Hum Mol Genet 18:R107–R112. 19297397 10.1093/hmg/ddp091

[acer14240-bib-0017] Jiang Y , He X , Howe PH (2012) Disabled‐2 (Dab2) inhibits Wnt/beta‐catenin signalling by binding LRP6 and promoting its internalization through clathrin. EMBO J 31:2336–2349. 22491013 10.1038/emboj.2012.83 PMC3364753

[acer14240-bib-0018] Kamel G , Hoyos T , Rochard L , Dougherty M , Kong Y , Tse W , Shubinets V , Grimaldi M , Liao EC (2013) Requirement for frzb and fzd7a in cranial neural crest convergence and extension mechanisms during zebrafish palate and jaw morphogenesis. Dev Biol 381:423–433. 23806211 10.1016/j.ydbio.2013.06.012

[acer14240-bib-0019] Knight RD , Schilling TF (2006) Cranial neural crest and development of the head skeleton. Adv Exp Med Biol 589:120–133. 17076278 10.1007/978-0-387-46954-6_7

[acer14240-bib-0020] Lee J , Cox BD , Daly CM , Lee C , Nuckels RJ , Tittle RK , Uribe RA , Gross JM (2012) An ENU mutagenesis screen in zebrafish for visual system mutants identifies a novel splice‐acceptor site mutation in patched2 that results in Colobomas. Invest Ophthalmol Vis Sci 53:8214–8221. 23150614 10.1167/iovs.12-11061 PMC3522441

[acer14240-bib-0021] Lovely CB , Swartz ME , McCarthy N , Norrie JL , Eberhart JK (2016) Bmp signaling mediates endoderm pouch morphogenesis by regulating Fgf signaling in zebrafish. Development 143:2000–2011. 27122171 10.1242/dev.129379 PMC4920158

[acer14240-bib-0022] Martik ML , Bronner ME (2017) Regulatory logic underlying diversification of the neural crest. Trends Genet 33:715–727. 28851604 10.1016/j.tig.2017.07.015 PMC5610108

[acer14240-bib-0023] McCarthy N , Wetherill L , Lovely CB , Swartz ME , Foroud TM , Eberhart JK (2013) Pdgfra protects against ethanol‐induced craniofacial defects in a zebrafish model of FASD. Development 140:3254–3265. 23861062 10.1242/dev.094938 PMC3931738

[acer14240-bib-0024] Neuhauss SC , Solnica‐Krezel L , Schier AF , Zwartkruis F , Stemple DL , Malicki J , Abdelilah S , Stainier DY , Driever W (1996) Mutations affecting craniofacial development in zebrafish. Development 123:357–367. 9007255 10.1242/dev.123.1.357

[acer14240-bib-0025] Obholzer N , Swinburne IA , Schwab E , Nechiporuk AV , Nicolson T , Megason SG (2012) Rapid positional cloning of zebrafish mutations by linkage and homozygosity mapping using whole‐genome sequencing. Development 139:4280–4290. 23052906 10.1242/dev.083931 PMC3478692

[acer14240-bib-0026] Pi X , Schmitt CE , Xie L , Portbury AL , Wu Y , Lockyer P , Dyer LA , Moser M , Bu G , Flynn EJ III , Jin SW , Patterson C (2012) LRP1‐dependent endocytic mechanism governs the signaling output of the bmp system in endothelial cells and in angiogenesis. Circ Res 111:564–574. 22777006 10.1161/CIRCRESAHA.112.274597 PMC3495066

[acer14240-bib-0027] Piotrowski T , Schilling TF , Brand M , Jiang YJ , Heisenberg CP , Beuchle D , Grandel H , van Eeden FJ , Furutani‐Seiki M , Granato M , Haffter P , Hammerschmidt M , Kane DA , Kelsh RN , Mullins MC , Odenthal J , Warga RM , Nusslein‐Volhard C (1996) Jaw and branchial arch mutants in zebrafish II: anterior arches and cartilage differentiation. Development 123:345–356. 9007254 10.1242/dev.123.1.345

[acer14240-bib-0028] Postlethwait JH (2006) The zebrafish genome: a review and msx gene case study. Genome Dyn 2:183–197. 18753779 10.1159/000095104

[acer14240-bib-0029] Riley EP , Infante MA , Warren KR (2011) Fetal alcohol spectrum disorders: an overview. Neuropsychol Rev 21:73–80. 21499711 10.1007/s11065-011-9166-x PMC3779274

[acer14240-bib-0030] Rochard L , Monica SD , Ling IT , Kong Y , Roberson S , Harland R , Halpern M , Liao EC (2016) Roles of Wnt pathway genes wls, wnt9a, wnt5b, frzb and gpc4 in regulating convergent‐extension during zebrafish palate morphogenesis. Development 143:2541–2547. 27287801 10.1242/dev.137000 PMC4958341

[acer14240-bib-0031] Rout UK (2006) Valproate, thalidomide and ethyl alcohol alter the migration of HTR‐8/SVneo cells. Reprod Biol Endocrinol 4:44 10.1186/1477-7827-4-44 16923192 PMC1592099

[acer14240-bib-0032] Rout UK , Dhossche JM (2010) Liquid‐diet with alcohol alters maternal, fetal and placental weights and the expression of molecules involved in integrin signaling in the fetal cerebral cortex. Int J Environ Res Public Health 7:4023–4036. 21139874 10.3390/ijerph7114023 PMC2996222

[acer14240-bib-0033] Saint‐Amant L , Sprague SM , Hirata H , Li Q , Cui WW , Zhou W , Poudou O , Hume RI , Kuwada JY (2008) The zebrafish ennui behavioral mutation disrupts acetylcholine receptor localization and motor axon stability. Dev Neurobiol 68:45–61. 17918238 10.1002/dneu.20569

[acer14240-bib-0034] Sarmah S , Muralidharan P , Marrs JA (2016) Embryonic ethanol exposure dysregulates BMP and notch signaling, leading to persistent atrio‐ventricular valve defects in Zebrafish. PLoS ONE ONE 11:e0161205. 10.1371/journal.pone.0161205 PMC4996461 27556898

[acer14240-bib-0035] Schilling TF , Piotrowski T , Grandel H , Brand M , Heisenberg CP , Jiang YJ , Beuchle D , Hammerschmidt M , Kane DA , Mullins MC , van Eeden FJ , Kelsh RN , Furutani‐Seiki M , Granato M , Haffter P , Odenthal J , Warga RM , Trowe T , Nusslein‐Volhard C (1996) Jaw and branchial arch mutants in zebrafish I: branchial arches. Development 123:329–344. 9007253 10.1242/dev.123.1.329

[acer14240-bib-0036] Sheehan‐Rooney K , Swartz ME , Zhao F , Liu D , Eberhart JK (2013) Ahsa1 and Hsp90 activity confers more severe craniofacial phenotypes in a zebrafish model of hypoparathyroidism, sensorineural deafness and renal dysplasia (HDR). Dis Models Mech 6:1285–1291. 10.1242/dmm.011965 PMC3759348 23720234

[acer14240-bib-0037] Shi J , Zhao W , Pan B , Zheng M , Si L , Zhu J , Liu L , Tian J (2017) Alcohol exposure causes overexpression of heart development‐related genes by affecting the histone H3 acetylation via BMP signaling pathway in cardiomyoblast cells. Alcohol Clin Exp Res 41:87–95. 27883221 10.1111/acer.13273

[acer14240-bib-0038] Smith SM (1997) Alcohol‐induced cell death in the embryo. Alcohol Health Res World 21:287–297. 15706739 PMC6827686

[acer14240-bib-0039] Solnica‐Krezel L , Schier AF , Driever W (1994) Efficient recovery of ENU‐induced mutations from the zebrafish germline. Genetics 136:1401–1420. 8013916 10.1093/genetics/136.4.1401 PMC1205920

[acer14240-bib-0040] Streissguth AP , Dehaene P (1993) Fetal alcohol syndrome in twins of alcoholic mothers: concordance of diagnosis and IQ. Am J Med Genet Part A 47:857–861. 10.1002/ajmg.1320470612 8279483

[acer14240-bib-0041] Swartz ME , Wells MB , Griffin M , McCarthy N , Lovely CB , McGurk P , Rozacky J , Eberhart JK (2014) A screen of zebrafish mutants identifies ethanol‐sensitive genetic loci. Alcohol Clin Exp Res 38:694–703. 24164477 10.1111/acer.12286 PMC3959233

[acer14240-bib-0042] Vangipuram SD , Grever WE , Parker GC , Lyman WD (2008) Ethanol increases fetal human neurosphere size and alters adhesion molecule gene expression. Alcohol Clin Exp Res 32:339–347. 18162078 10.1111/j.1530-0277.2007.00568.x

[acer14240-bib-0043] Veth KN , Willer JR , Collery RF , Gray MP , Willer GB , Wagner DS , Mullins MC , Udvadia AJ , Smith RS , John SW , Gregg RG , Link BA (2011) Mutations in zebrafish lrp2 result in adult‐onset ocular pathogenesis that models myopia and other risk factors for glaucoma. PLoS Genet 7:e1001310. 21379331 10.1371/journal.pgen.1001310 PMC3040661

[acer14240-bib-0044] Wada N , Javidan Y , Nelson S , Carney TJ , Kelsh RN , Schilling TF (2005) Hedgehog signaling is required for cranial neural crest morphogenesis and chondrogenesis at the midline in the zebrafish skull. Development 132:3977–3988. 16049113 10.1242/dev.01943

[acer14240-bib-0045] Walker MB , Kimmel CB (2007) A two‐color acid‐free cartilage and bone stain for zebrafish larvae. Biotech Histochem 82:23–28. 17510811 10.1080/10520290701333558

[acer14240-bib-0046] Warren KR , Li TK (2005) Genetic polymorphisms: impact on the risk of fetal alcohol spectrum disorders. Birth Defects Res A Clin Mol Teratol 73:195–203. 15786496 10.1002/bdra.20125

[acer14240-bib-0047] Wentzel P , Eriksson UJ (2009) Altered gene expression in neural crest cells exposed to ethanol in vitro. Brain Res 1305(Suppl):S50–S60. 19703426 10.1016/j.brainres.2009.08.057

[acer14240-bib-0048] Willems B , Tao S , Yu T , Huysseune A , Witten PE , Winkler C (2015) The Wnt co‐receptor Lrp5 is required for cranial neural crest cell migration in zebrafish. PLoS ONE ONE 10:e0131768. 10.1371/journal.pone.0131768 PMC4486457 26121341

[acer14240-bib-0049] Woods IG , Wilson C , Friedlander B , Chang P , Reyes DK , Nix R , Kelly PD , Chu F , Postlethwait JH , Talbot WS (2005) The zebrafish gene map defines ancestral vertebrate chromosomes. Genome Res 15:1307–1314. 16109975 10.1101/gr.4134305 PMC1199546

